# Physical Activity as a Determinant of Successful Aging over Ten Years

**DOI:** 10.1038/s41598-018-28526-3

**Published:** 2018-07-12

**Authors:** Bamini Gopinath, Annette Kifley, Victoria M. Flood, Paul Mitchell

**Affiliations:** 1 Centre for Vision Research, Department of Ophthalmology and The Westmead Institute for Medical Research, The University of Sydney, Sydney, Australia; 2 Faculty of Health Sciences, The University of Sydney, Sydney, Australia; 3 St Vincent’s Hospital, Sydney, Australia

## Abstract

We aimed to examine the temporal association between physical activity and successful aging. The analyses involved 1,584 adults aged 49 + years living west of Sydney (Australia), who did not have cancer, coronary artery disease and stroke at baseline and who were followed over 10 years. Participants provided information on the performance of moderate or vigorous activities and walking exercise and this was used to determine total metabolic equivalents (METs) minutes of activity per week. Successful aging status was determined through interviewer-administered questionnaire and was classified as the absence of: depressive symptoms, disability, cognitive impairment, respiratory symptoms and systemic conditions (e.g. cancer, coronary artery disease). 249 (15.7%) participants (mean age 59.9 ± 6.1) had aged successfully 10 years later. After multivariable adjustment; older adults in the highest level of total physical activity (≥5000 MET minutes/week; n = 71) compared to those in the lowest level of total physical activity (<1000 MET minutes/week; n = 934) had 2-fold greater odds of aging successfully than normal aging, odds ratio, OR, 2.08 (95% confidence intervals, CI, 1.12–3.88). Older adults who engaged in high levels of total physical activity, well above the current recommended minimum level had a greater likelihood of aging successfully 10 years later.

## Introduction

Successful aging is specified as a multi-domain concept that comprises and transcends good health, and is made up of a wide spectrum of biopsychosocial factors; for example, Rowe and Kahn previously defined successful aging as not suffering from chronic diseases, having optimal social engagement and mental health, as well as a lack of physical disability^1^. It has been posited that this type of a multi-domain approach of assessing aging status, rather than focussing on risk factors for individual health outcomes, such as disability or functioning could be more useful^2,3^.

Numerous studies have found that physical activity decreases the risk of many chronic diseases and increases longevity^4–7^. However, the association between physical activity and successful aging (determined as a multidimensional concept) has shown heterogeneity across studies^2^. Some studies have shown either a lack of or a weak independent association between physical activity and successful aging^6,8,9^; however, other cohort studies as well as systematic reviews have shown that higher levels of physical activity (based on frequency of participation or energy expenditure in a range of household, leisure, or exercise activity) was associated with aging successfully^2,7,10^.

A clear understanding of the associations between behavioral determinants, such as physical activity, and successful aging is essential in the preparation of effective measures of health promotion and disease/disability prevention in global planning for the well-being of older adults^7^. Therefore, in our cohort study of adults aged 49+ years at baseline we aimed to investigate whether total physical activity is independently associated with successful aging, which was defined as not experiencing disability and chronic disease (coronary artery disease, stroke, diabetes, cancer), having good mental health and functional independence, and reporting optimal physical, respiratory and cognitive function during 10 years of follow-up. For comparison, we also examined the association between levels of physical activity and 10-year mortality risk in this population.

## Methods

### Study population

**Figure 1:**
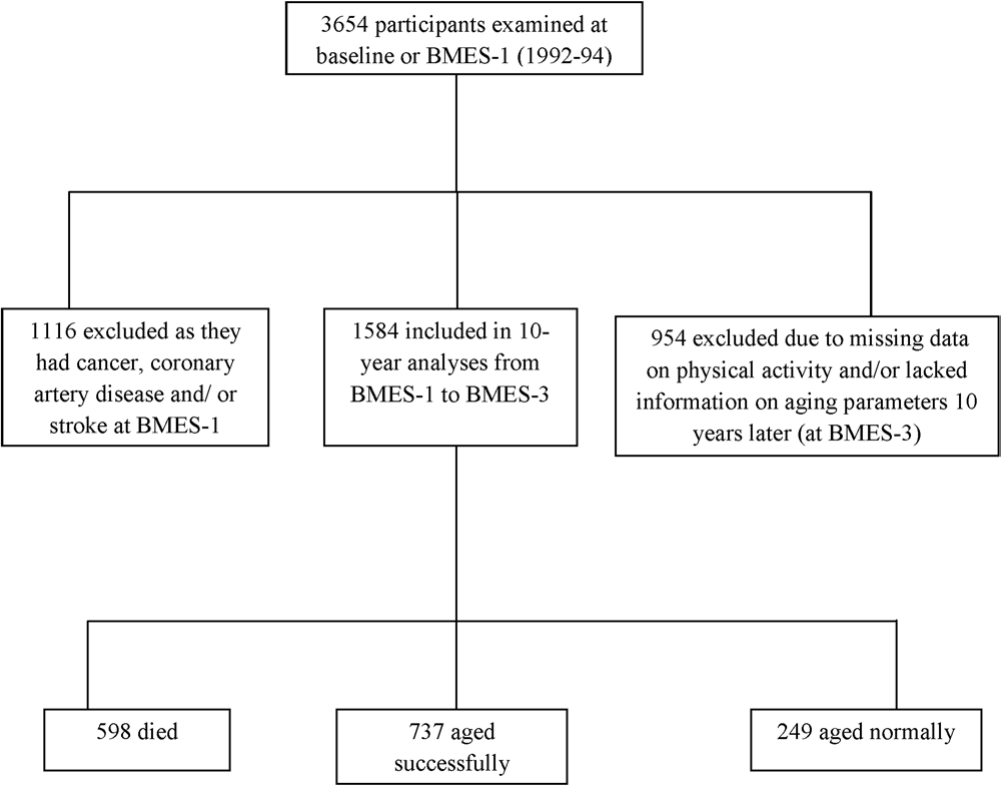
Flowchart showing study participation in the Blue Mountains Eye Study.

The Blue Mountains Eye Study (BMES) is a population-based study of common eye conditions and a range of other health outcomes in a suburban population, west of Sydney, Australia. Study methods have been previously described^11^. At baseline, 3654 residents aged >49 years were examined during 1992-4 (BMES-1, 82.4% participation rate). Re-examinations of surviving baseline participants were conducted after 5- (1997-9, BMES-2), 10- (2002-4, BMES-3), and 15 years (2007-9, BMES-4) at which 2334 (75.1% of survivors), 1952 participants (75.6% of survivors) and 1149 (55.4% of survivors) were followed-up, respectively (Fig. 1). Ethical approval was obtained from the University of Sydney and the Western Sydney Area Human Ethics Committees, and written informed consent was obtained from all study participants at each follow-up. All study methods were performed in accordance with the relevant guidelines and regulations.

### Assessment of physical activity

The physical activity questionnaire used in the BMES was based on the validated International Physical Activity Questionnaire (IPAQ)^12^. Participants provided information on the performance of moderate or vigorous activities and walking exercise^13^, which were used to determine metabolic equivalents (METs) over 1 week^12^. Participants answered either yes/ no/ don’t know to the following questions: (1) In the last 2 weeks did you walk for recreation or exercise for at least 10 minutes continuously? (2) In the past 2 weeks did you do any vigorous activity or exercise which made you breathe harder or puff and pant? (e.g. carrying loads, heavy gardening, chopping wood, labouring – at home, during work or anywhere else) and (3) In the past 2 weeks did you do any other leisure time physical activities that you haven’t already mentioned? (e.g. more moderate activities such as lawn bowls, gardening). Participants who answered ‘yes’ were then asked how many times in the last 2 weeks and the estimated time (in hours and minutes) that they spent walking, doing vigorous activity and/or leisure time activities. MET minutes of activity per week calculations were based on the IPAQ scoring protocol i.e. were calculated as duration × frequency per week × MET intensity, which were summed across activity domains to produce a weighted estimate of total physical activity from all reported activities per week^12^. Information on METs was obtained at baseline or BMES-1 was analyzed for the current report.

### Assessment of aging and survivial

We assessed chronic diseases throughout the 10-year follow-up (i.e. from BMES-1 to BMES-3). Medical history was determined by interviewer-administered questionnaire at each visit. The normal aging group were all participants who were alive 10 years later, but who were not deemed as successful agers^2^. Among surviving participants at 10-year follow-up (aged 60+ years), we used a definition similar to that used by *Sabia et al*.^2^, which classified successful agers as filling the following criteria: absence of stroke, coronary artery disease, angina, acute myocardial infarction (AMI), cancer, or diabetes; optimal cognitive, physical, mental, respiratory and vascular function; and the lack of disability; and being functionally independent^14,15^.

To establish participants who died after BMES-1, demographic data including surname, first and second names, date of birth and sex of the participants were cross-matched with Australian National Death Index (NDI) data^16^. High sensitivity and specificity for cardiovascular mortality (92.5% and 89.6%, respectively) for NDI data has been previously reported^17^. The census cut-off for deaths was late December 2004 (i.e. 10-year follow-up since BMES-1 or the baseline study)^14,15^.

### Assessment of potential confounders

Participants reported who they lived with (alone or with e.g. child, partner, friend) and marital status (married, widowed, divorced, or never married). Body mass index was calculated as weight divided by height squared (kg/m^2^). Participants reported whether they had never smoked or were past or current smokers. Current smoking included those who reported quitting smoking in the past year.

### Statistical Analysis

Statistical analyses were performed using SAS 9.3 software (SAS Institute, Cary, NC, USA). Study factor was physical activity (in MET minutes/week) and aging status the study outcome. Multivariable polytomous logistic regression analyses of physical activity and the outcome of aging status (successful aging, normal aging, and death) used the generalized logit link and adjusted for: age, sex, marital status, living status, smoking, and weight status. The association between physical activity at baseline and aging status at 10-year follow-up was analysed by tertiles of physical activity, using the lowest tertile of activity as the reference group, and for each 1000 MET minutes/week increase in baseline activity. Study characteristics of participants at baseline who were the re-examined 10 years later were compared using χ^2^-tests and general linear model. We also examined cross-lagged panel models for physical activity (in METs), functional independence and the presence of specific illnesses (e.g. cancer, coronary artery disease) at baseline and 10-year follow-up using MPLUS weighted least squares estimation with missing data. This additional analysis allowed us to further examine how physical activity, physical function and specific illnesses/comorbidities were related cross-sectionally and over time.

## Results

Over the 10 years of follow-up, there were 249/15.7% (mean age: 59.9 ± 6.1), 737/46.5% (mean age: 62.0 ± 7.4), and 598/37.8% (mean age: 71.3 ± 9.5) participants who were successful agers, normal agers or who died, respectively (Fig. 1). At baseline, participants who aged successfully compared to those who were classified as normal agers or had died were more likely to be younger, married and physically active, but less likely to smoke or live alone at baseline (Table 1). We tested for effect modification by sex, BMI and smoking status on aging status; and no significant interactions were observed between sex, BMI or smoking status and physical activity on aging status (all p > 0.05). Moreover, the number of participants who were obese or who were currently smoking were too small and thus, we would not be able to meaningfully examine associations between physical activity and healthy aging within these groups.

**Table 1:** Study characteristics of participants at baseline stratified by aging status (n = 1584).

Characteristics	Normal aging (n = 737)	Successful aging (n = 249)	Died (ns = 598)	P-value
Age, *yrs*	62.0 (7.4)	59.9 (6.1)	71.3 (9.5)	<0.0001
Male sex	275 (37.3)	105 (42.2)	308 (51.5)	<0.0001
Married	494 (67.0)	191 (76.7)	353 (59.3)	<0.0001
Lives alone	158 (21.5)	46 (18.6)	195 (33.1)	<0.0001
Current smoking	103 (14.2)	20 (8.2)	103 (17.9)	0.002
Overweight/obese	127 (17.4)	33 (13.3)	89 (15.2)	0.26
Physical activity, METs	1335.6 (2055)	1798.2 (3046)	1127.4 (1871)	<0.0001

METs – Metabolic Equivalents minutes of activity per week. Data are presented as means ± SD or n (%).

Table 2 shows that participants who engaged in ≥5000 MET minutes/week compared to those who reported <1000 MET minutes/week (reference group) at baseline, had a 2-fold higher likelihood of successful aging rather than normal aging after accounting for potential confounders. The overall continuous trend per 1000 unit increase in baseline physical activity was also significantly associated with successful aging at the 10-year follow-up: multivariable-adjusted OR 1.08, (95% CI 1.02–1.14), p = 0.008. Physical activity was not associated with 10-year mortality risk in our cohort (Table 2). Further, there were no significant associations or trends observed between increasing tertiles of MET minutes/week and odds of successful aging or mortality risk in comparison with the normal aging group (data not shown).

**Table 2:** Association between baseline physical activity and aging status 10 years later in the Blue Mountains Eye Study.

Physical activity (METs)	Normal aging (n = 737)		Successful aging (n = 249)		Died (n = 598)	
	n (%)	OR (95% CI)^a^	n (%)	OR (95% CI)^a^	n (%)	OR (95% CI)^a^
<1000 (n = 934)	419 (44.9)	1.0 (reference)	136 (14.6)	1.0 (reference)	379 (40.6)	1.0 (reference)
1000–1999 (n = 369)	181 (49.1)	1.0 (reference)	56 (15.2)	0.96 (0.67–1.39)	132 (35.8)	0.92 (0.67–1.27)
2000–2999 (n = 119)	63 (52.9)	1.0 (reference)	23 (19.3)	1.04 (0.61–1.75)	33 (27.7)	0.65 (0.39–1.10)
3000–3999 (n = 57)	29 (50.9)	1.0 (reference)	8 (14.0)	0.84 (0.37–1.91)	20 (35.1)	0.84 (0.43–1.66)
4000–4999 (n = 34)	14 (41.2)	1.0 (reference)	6 (17.7)	1.56 (0.57–4.22)	14 (41.2)	1.10 (0.45–2.70)
≥5000 (n = 71)	31 (43.7)	1.0 (reference)	20 (28.2)	**2.08 (1.12–3.88)**	20 (28.2)	0.77 (0.40–1.47)

^a^Adjusted for age, sex, marital status, living status, smoking, and body mass index.

We also examined cross-lagged panel models for physical activity, functional independence and the presence of specific illnesses (e.g. cancer, coronary artery disease) at baseline and 10-year follow-up to help address some of the temporality issues. In models that included inter-relationships between each of these three variables within and across time points (baseline and 10 years), physical activity at baseline (p = 0.027) and presence of specific illnesses at baseline (p < 0.001) were both significantly associated with functional independence at 10 years. Specific illnesses at baseline (p = 0.2) and independence at baseline (p = 0.3) were not significantly associated with physical activity at 10 years. Baseline status was strongly associated with status at 10 years for each of the three measures (p < 0.0001 in all three cases). Supplementary analysis was conducted, which involved only the subgroup of 1353 participants who were free of chronic diseases at baseline and also free of disability (e.g. did not have a walking disability) and were functionally independent (e.g. did not require support from family and friends and did not use community support services such as meals on wheels) at BMES-1 (baseline). After multivariable adjustment, the association between total physical activity and successful aging became marginally non-significant (p = 0.07) i.e. participants who engaged in ≥ 5000 MET minutes/week compared to those who reported <1000 MET minutes/week (reference group) at baseline, had the following odds of successful aging rather than normal aging: OR 1.82 (95% CI 0.94–3.51). However the overall continuous trend per 1000 unit increase in physical activity was still significantly associated (p = 0.03) with successful aging: OR 1.07 (95% CI 1.01–1.14).

## Discussion

With the aging demographics of most countries, a major challenge is to consider how to increase the quality and years of healthy life^7^. Our findings from this community-based study has moved the research forward in this area by showing an independent and positive association between total physical activity and a multidimensional concept of successful aging over 10 years of follow-up, in an initially disease-free population. Specifically, we show that older adults who engaged in higher levels total physical activity (≥5000 MET minutes/week) at baseline were 2-fold more likely to be disease-free and fully functional, that is, having aged successfully 10 years later.

Our finding of a positive association between physical activity levels and successful aging is in agreement with the existing literature showing that physical activity might be an important parameter in enabling people to age successfully^2,7,10,18^. Moreover, a systematic review^7^ found that the effect sizes for the association of successful or healthy aging with high levels of physical activity ranged from 1.27 to 3.09, which is in line with the observed estimate of 2.08 observed for the association between the highest levels of physical activity and successful aging in our cohort.

The mechanisms underlying the influence of high levels of physical activity on aging status remain unclear. One speculated mechanism could involve inflammatory pathways^19^. Regular physical activity is associated with sustained lower levels of inflammatory markers in older adults^19^. Further, low-grade inflammation has been linked to many of the components of successful aging, including chronic disease^20,21^ depression^22^ cognitive decline^23^, sarcopenia and disability^21^. It is also well known that physical activity impacts on telomere length and may slow down the aging process^24,25^.

Epidemiological data from the current study also aligns with findings of a recent systematic review and dose-response meta-analysis^4^, which showed that a higher level of total physical activity is strongly associated with a lower risk of cancer, diabetes, heart disease and stroke with most health gains occurring at a total activity level of 3000–4000 MET minutes/week. These previous findings as well as our data suggest that levels of physical activity need to be several times higher than the WHO recommended minimum level of 600 MET minutes/week for larger reductions in the risk of chronic diseases^4^, and consequently to achieve a successful aging status in the longer term. Some older adults may not be able to engage in vigorous activity or very high levels of physical activity^4^, however, at the population level there is a spectrum of capability to perform physical activity at an older age^4,5^. Therefore, it has been suggested that a population strategy be adopted where the population distribution of physical activity is shifted rather than focusing solely on high-risk subgroups in the community^4,26^. The population strategy to promote physical activity among older adults should be to get those who are inactive to do some physical activity and those who currently only engage in moderate activity to incorporate more vigorous activity if feasible^5^.

It is also important to acknowledge that the definition of successful aging used in our study does not take into account non-normative, individual trajectories of successful development in older age^27^. For instance the life-span model of Selective Optimization with Compensation model (SOC-model) developed by *Baltes et al*.^28^, specifies that aging may be best defined as a heterogeneous process with several different pathways and (successful) outcomes, that is, instead of solely defining the end points it considers that overall process of successful aging^27^. Therefore, future research on the influence of physical activity in the context of a heterogeneous process (e.g. SOC model) with many different pathways that may all result in the maintenance of life satisfaction in advanced age^27^ could be valuable^18^. Strengths of this study include its prospective design, long-term follow-up of a stable population-based cohort, and use of an extensive definition of successful aging. We also aimed to minimize potential confounding by adjusting for key covariates such as age, sex, weight, smoking, marital status etc. However, our study has some limitations. We only had self-reported measures of physical activity rather than objective measurements of physical activity levels. Nevertheless, the costs, logistics and expertise required to obtain accelerometer-measured METs can be prohibitive for such a large sample of participants. However, self-reported physical activity in older adults has been shown to overestimate actual activity^19^ and for this reason, we might have underestimated the magnitude of association between physical activity and aging status in our study. Similarly, aging outcomes were not objectively measured (e.g. respiratory function); hence, we cannot disregard possible measurement errors^15^. Finally, while our definition of successful aging was comprehensive, we were still not able to account for important social variables such as loneliness and financial stresses^18^, hence, the proportion of BMES participants aging successfully could be lower than that reported.

In conclusion, our study has shown that a higher level of physical activity increases the likelihood of surviving an additional 10 years free of chronic diseases, cognitive impairment and functional disability. These findings underscore the importance of placing greater attention and investments in public health interventions aiming to promote physical activity participation among older adults living in the community.
